# Onvansertib and Navitoclax Combination as a New Therapeutic Option for Mucinous Ovarian Carcinoma

**DOI:** 10.3390/ijms26020472

**Published:** 2025-01-08

**Authors:** Serena Petrella, Marika Colombo, Mirko Marabese, Chiara Grasselli, Andrea Panfili, Michela Chiappa, Valentina Sancisi, Ilaria Craparotta, Maria C. Barbera, Giada A. Cassanmagnago, Marco Bolis, Giovanna Damia

**Affiliations:** 1 Laboratory of Gynecological Preclinical Oncology, Department of Experimental Oncology, Istituto di Ricerche Farmacologiche Mario Negri IRCCS, 20156 Milan, Italy; serena.petrella@marionegri.it (S.P.); michela.chiappa@marionegri.it (M.C.); 2 Laboratory of Molecular Pharmacology, Department of Experimental Oncology, Istituto di Ricerche Farmacologiche Mario Negri IRCCS, 20156 Milan, Italy; marika.colombo@marionegri.it (M.C.); mirko.marabese@marionegri.it (M.M.); 3 Laboratory of Immunopharmacology, Department of Experimental Oncology, Istituto di Ricerche Farmacologiche Mario Negri IRCCS, 20156 Milan, Italy; chiara.grasselli@marionegri.it (C.G.); andrea.panfili@marionegri.it (A.P.); 4 Translational Research Laboratory, Azienda Unità Sanitaria Locale-IRCCS di Reggio Emilia, 42123 Reggio Emilia, Italy; valentina.sancisi@ausl.re.it; 5 Computational Oncology Unit, Department of Experimental Oncology, Istituto di Ricerche Farmacologiche Mario Negri IRCCS, 20156 Milan, Italy; ilaria.craparotta@marionegri.it (I.C.); mariachiara.barbera@marionegri.it (M.C.B.); giadaandrea.cassanmagnago@marionegri.it (G.A.C.); marco.bolis@marionegri.it (M.B.)

**Keywords:** mucinous ovarian cancer, CRISPR/Cas9 screening, BCL2L2, navitoclax, onvansertib

## Abstract

Mucinous epithelial ovarian cancer (mEOC) is a rare subtype of epithelial ovarian cancer, characterized by poor responses to standard platinum-based chemotherapy. Polo-like kinase 1 (PLK1) is a key regulator of mitosis and cell cycle progression and its inhibition has been recently identified as a target in mEOC. In this study, we aimed to identify further therapeutic targets in mEOC using a CRISPR/Cas9 library targeting 3015 genes, with and without treatment with onvansertib, a PLK1 inhibitor. We identified twelve genes associated with cell survival (*ZC2HC1C*, *RPA2*, *KIN17*, *TUBG1*, *SMC2*, *CDC26*, *CDC42*, *HOXA9*, *TAF10*, *SENP1*, *MRPS31*, and *COPS2*) and three genes (*JUND*, *CARD9*, and *BCL2L2*) in synthetic lethality with onvansertib treatment. We validated that *SENP1* downregulation is important for the growth of mEOC cells through esiRNA interference and the use of a pharmacological inhibitor Momordin Ic. The downregulation of *CARD9* and *BCL2L2* combined with subtoxic doses of onvansertib interfered with mEOC cell growth. Interestingly, the combination of navitoclax, an inhibitor of BcL2 family members including BCL2L2, was synergistic in all four of the mEOC cell lines tested and substantially induced cell death through apoptosis. These data support the use of a combination of navitoclax and onvansertib as a new therapeutic strategy for mEOC.

## 1. Introduction

Epithelial ovarian cancer (EOC) is the second most common cause of death from a gynecological cancer. Globally there are 313,959 new cases (1.6% of all cancers) and 207,252 deaths (2.1% of all cancer deaths) annually [1]. Five different histotypes of EOC have been recognized, each with unique risk factors, molecular profiles, and clinical prognosis. The high-grade serous histotype accounts for 70% of EOCs, low-grade serous accounts for 10%, endometrioid for 10%, clear cell for 5%, and mucinous (mEOC) for less than the 3% [2]. mEOC is in fact the rarest EOC histotype, with a median age at diagnosis of 50–54 years. Typical mEOC presents at an early stage (~80% stage I, ~10% stage II, ~10% stage III, ~10% stage IV) [3]. When diagnosed at early stages, mEOC has a good prognosis, but when diagnosed at advanced stages, the prognosis is dismal, with the cancer responding poorly to standard platinum-based regimens [4]. In fact, the median survival of stage III/IV disease is less than 15 months, compared to 41 months for high-grade serous ovarian carcinoma [5]. Given these poor outcomes, new therapeutic strategies are needed. For new therapies, identifying effective drug targets is critical.

PLK1 is a member of the well-conserved serine/threonine kinase family, which plays a key role in the progression of mitosis, in G2/M checkpoint regulation, DNA damage replication, stress response, and cell death pathways [6,7,8,9]. PLK1 is overexpressed in various cancers, including invasive breast carcinoma [10], liver carcinoma [11], colon adenocarcinoma [12], lung squamous cell carcinoma [13], prostate adenocarcinoma [14], gastric cancer [15], ovarian cancer [16], and melanoma [17]. Tumor PLK1 levels correlate with tumorigenicity, aggressiveness, and poor prognosis for different types of tumors [18]. Different studies have shown that inhibiting PLK1 using RNAi or small molecule inhibitors interferes with cancer cell proliferation [19,20,21,22] and this has fostered the development of PLK1 inhibitors (PLK1is) as new anti-cancer drugs [23]. Among the PLK1is developed, onvansertib (NMS-P937) is a highly selective, orally available drug discovered through high-throughput screening [24]. Its selectivity is due to its ability to form a specific hydrogen bond with the side chain of Glu140 in PLK1, which is absent in PLK2 and PLK3 due to the presence of histidine. Onvansertib is active against various carcinomas and hematologic cell lines and has been found to cause tumor regression in vivo [24,25]. In a phase 1b/2 clinical study, in patients with *KRAS*-mutated metastatic colorectal cancer (mCRC), it was shown to be safe and effective when combined with chemotherapy [26]. Onvansertib received Fast Track Designation from the FDA in 2020 for *KRAS-*mutated mCRC treatment, and recent positive data from Cardiff Oncology highlights its potential in ongoing trials for metastatic pancreatic ductal adenocarcinoma (mPDAC) and small-cell lung cancer (SCLC) [27]. Interestingly, onvansertib showed antitumoral activity in combination with paclitaxel in EOC preclinical models, including mEOC [28,29].

Recently, onvansertib has also been reported to overcome resistance to olaparib (a poly-ADP–ribose–polymerase inhibitor) in models of patient-derived xenografts of high-grade serous ovarian carcinomas [30].

CRISPR/Cas9 can precisely target genes for deletion, mutation, or insertion, making it a versatile and powerful tool for drug development and biomedical research [31]. Cas9-mediated DNA cleavage can introduce frameshift mutations that disrupt open reading frames, leading to gene knockouts [31]. Recent advances in high-throughput CRISPR/Cas9 screening have facilitated the synthesis of oligonucleotides to construct plasmid libraries, which are delivered to target cells by viral transduction. This has enabled researchers to identify genes crucial for cell survival and, in conjunction with a specific treatment, it can reveal genes involved in drug sensitivity and resistance and possibly new synthetic lethal interactions [32]. For example, this type of approach has allowed for the identification of *PCMT1* as key gene in ovarian cancer metastasis [33], and highlights the importance of the Hippo pathway in drug-resistant EGFR-mutant lung cancer [34].

Considering the poor prognosis of mEOC, new therapeutic targets are needed. In addition, having identified PLK1 as a new therapeutic target, the finding of lethal pharmacological interactors for PLK1 might offer a valuable therapeutic strategy.

We therefore carried out CRISPR/Cas9 screening using the lentiviral Bassik Human CRISPR Deletion Library—Apoptosis and Cancer on a mucinous cell line, in the presence or absence of a subtoxic concentration of onvansertib. Our objective was to identify new targets in mEOC and potential targets eligible for combination with onvansertib

## 2. Results

### 2.1. Generation of Cas9 mEOC Cell Lines

**Figure 1:**
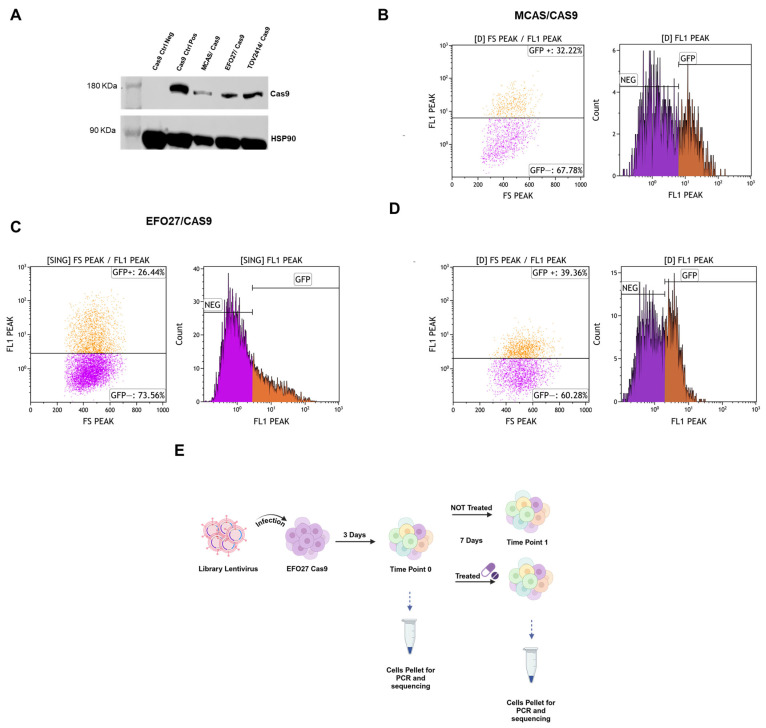
Screening experiment and Cas9 in mEOC cell lines. (**A**) Western blot analysis of Cas9 in mEOC cells. (**B**–**D**) Flow cytometric analyses of GFP positive (GFP+) and negative (GFP−) cells after lentivirus transduction of GFP plasmid carrying gRNA for GFP in MCAS/Cas9 (**B**), EFO27/Cas9 (**C**) and TOV2414/Cas9 (**D**). (**E**) Workflow of screening experiment.

In order to create a gRNAs library, we first generated mEOC cell lines expressing Cas9. We used three different cell lines: MCAS, EFO27, and TOV2414. Cells were transduced using lentivirus carrying the *Cas9* gene. After transduction, Cas9 expression was evaluated by Western blot (Figure 1A) and the activity of the Cas9 enzyme was assessed by a GFP assay, which exploits the plasmid carrying the sequence of GFP, a gRNA for GFP and resistance to puromycin. Cas9 activity is correlated with the percentage of GFP-negative cells [35]. All the mEOC cell lines had substantial GFP-negative population: 67.78% for MCAS/Cas9, 73.56% for EFO27/Cas9, and 60.27% for TOV2414/Cas9 (Figure 1B–D).

### 2.2. CRISPR Screening in EFO27/Cas9

**Figure 2:**
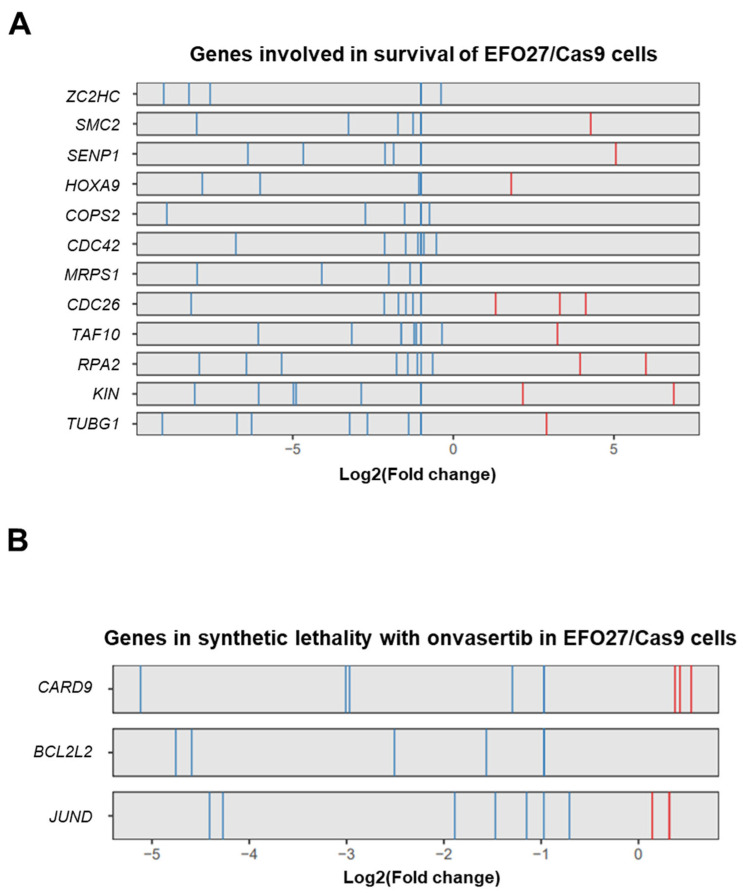
Genes identified by CRISPR/Cas9 screening in EFO27/Cas9. (**A**) There were 12 genes with an FDR < 0.05 compared to the not treated groups T1 and T0. These are the genes that, if depleted, lead to cell death. (**B**) Comparing T1-treated with T1-untreated genes, the selection based on the FDR < 0.1 of individual sgRNAs allowed for the identification of three genes with potential synthetic lethality with onvansertib. For each indicated gene, blue lines represent the gRNAs that were downregulated, while the red lines are the gRNAs that were upregulated. CRISPR-seq data were processed using the MAGeCK pipeline [36] in the MAGeCKFlute R package [37].

To identify genes that could be new therapeutic targets for mEOC, alone or combined with onvansertib, we carried out CRISPR/Cas9 screening on the EFO27 cell lines. The experimental setting is detailed in the Section 4 and summarized in Figure 1E. A bioinformatic analysis of decreased sgRNAs at T1 compared to T0 led to the identification of 12 genes potentially associated with cell survival, with a false discovery rate (FDR) < 0.05 (Figure 2A): *ZC2HC1C*, *RPA2*, *KIN*, *TUBG1*, *SMC2*, *CDC26*, *CDC42*, *HOXA9*, *TAF10*, *SENP1*, *MRPS31*, *COPS2*. A comparative analysis of cells treated with onvansertib at T1 versus untreated cells led to the identification of three genes with differentially expressed sgRNAs, suggesting a possible synergistic effect between onvansertib and these genes (Figure 2B).

### 2.3. Validation of Genes Involved in Survival

Among the genes that were found to be important for EFO27 cell survival, *KIN17* and *SENP1* were selected for further validation. *KIN17* was chosen because it was one of the most significant genes, and it is involved in DNA damage repair, a mechanism that we know is crucial in ovarian cancer [38]. *SENP1* was chosen due to its involvement in different pathways, and it may have a role in responses to chemotherapy in ovarian cancer [39].

**Figure 3:**
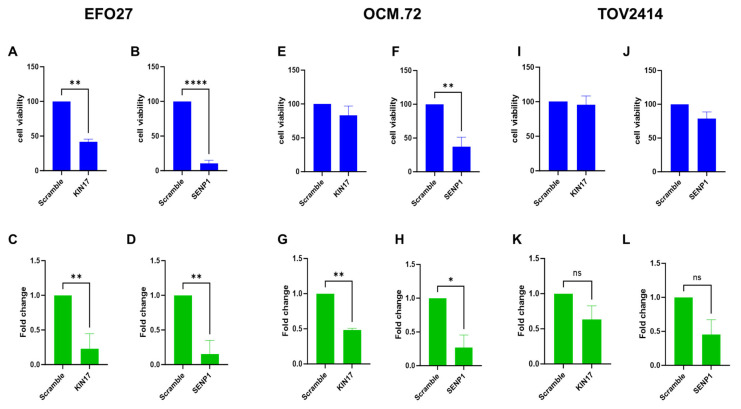
Effects of esiRNA *KIN17* and *SENP1* transfection. Cell viability of EFO27 (**A**,**B**), OCM.72 (**E**,**F**), and TOV2414 (**I**,**J**) cells transfected with esiRNA for *KIN17* and *SENP1*. Data are the mean ± SD of at least three independent experiments run in triplicate, and are expressed as the percentage on scramble-transfected cells. After 72 h following transfection, RNA was extracted from cells transfected with target and scramble esiRNAs and analyzed to evaluate the expression of targeted genes. The relative-fold change in the selected genes after transfection with the selected esiRNA compared to scramble esiRNA-transfected cells. Mean ± SD of at least three independent experiments (**C**,**D**,**G**,**H**,**K**,**L**). ns: not significant; *: *p* < 0.05; **: *p* < 0.01; ****: *p* < 0.0001

To validate the results from the screening, we transiently transfected EFO27 with specific esiRNAs. The downregulation of *KIN17* and *SENP1* (Figure 3C,D) resulted in a significant decrease in cell viability, compared to scramble siRNA-transfected cells (Figure 3A,B). The downregulation of *KIN17* was confirmed by Western blot analysis (Supplementary Figure S1A). Interestingly, the downregulation of both genes was associated with the induction of apoptosis (Supplementary Figure S1B). When the same experiments were conducted in OCM.72 cells, KIN17 downregulation (Figure 3G) did not affect cell growth (Figure 3E). However, SENP1 downregulation (Figure 3H) inhibited the cell growth of OCM.72 (Figure 3F). In TOV2414 cells, we were unable to achieve effective downregulation of either gene (Figure 3K,L).

When we looked for commercially available inhibitors, we could only find an inhibitor of SENP1: Momordin Ic. This is a natural triterpenoid saponin used in various Chinese natural medicines; it has an antioxidant capacity, inhibits ethanol-induced gastric mucosal lesions, accelerates gastrointestinal transit, and prevents glucose-induced blood sugar increases, with some undefined anti-cancer properties [40]. We tested its cytotoxic activity in all four different mucinous cell lines available. We treated the cells with Momordin Ic at concentrations between 0 and 30 µM for five days, and analyzed cell viability using the CellTiter assay. The IC50s were 13.19 ± 1.43 µM for EFO27, 10.68 µM ± 1.08 for OCM.72, 33.01 µM ± 10.53 for MCAS, and 24.3 µM ± 13.09 for TOV2414 (Supplementary Figure S2).

### 2.4. Validation of Genes in Synthetic Lethality with Onvansertib

**Figure 4:**
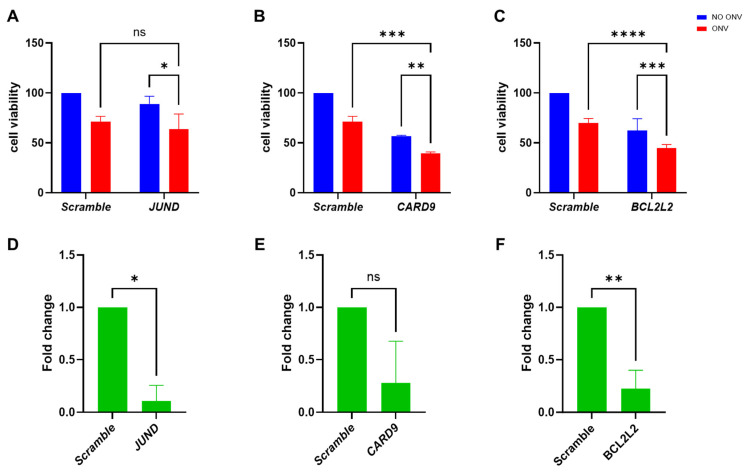
Effects of esiRNA *JUND*, *CARD9* and *BCL2L2* transfection in EFO27. Cellular viability of EFO27 cells transfected with the esiRNAs *JUND* (**A**), CARD9 (**B**), and BCL2L2 (**C**) with or without onvansertib treatment. Data are the mean ± SD of at least two independent experiments and are expressed as percentage of negative control cells. After 72 h following transfection, RNA was extracted from cells transfected with target and scramble esiRNAs and analyzed to evaluate the expression of targeted genes (**D**–**F**). Data expressed as relative-fold change in target compared to scramble. Results are the mean ± SD of at least two independent experiments. ONV: onvansertib. ns: not significant; *: *p* < 0.05; **: *p* < 0.01; ***: *p* < 0.001; ****: *p* < 0.0001

We then validated the genes resulting from the screening in combination with onvansertib. Transfection with the esiRNA of *JUND*, *CARD9*, and *BCL2L2*, combined with a subtoxic dose of onvansertib (150 nM), resulted in significant reduction in cell viability as compared to single onvansertib treatment and single gene downregulation (Figure 4A–C). The gene downregulations were confirmed by qRT-PCR (Figure 4D–F).

### 2.5. Synergistic Effect of Navitoclax and Onvansertib in mEOC Celles

We then looked for chemical inhibitors, and focused on navitoclax (ABT263), an orally available inhibitor targeting anti-apoptotic B-cell lymphoma 2 proteins (BCL-XL, BCL-2, BCL-W) that has antitumor activity and can induce apoptosis [41], as no specific inhibitors of BCL2L2 are available yet.

**Figure 5:**
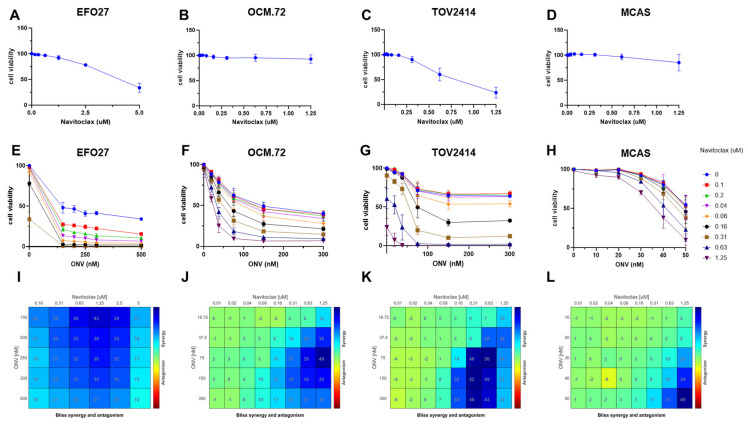
Combination of navitoclax and onvansertib on mEOC cells. Dose–response curve for navitocax in EFO27 (**A**), OCM.72 (**B**), TOV2414 (**C**), and MCAS (**D**) cells. Combination of onvansertib and navitoclax in EFO27 (**E**), OCM.72 (**F**), TOV2414 (**G**), and MCAS (**H**) cells; the blue curve is onvansertib alone, and the other curves are the combinations with different concentrations of navitoclax. Data are the mean ± SD of at least two independent experiments and are expressed as the percentage of control untreated cells. (**I**–**L**) Drug synergy is indicated by blue squares in the Bliss Synergy Heatmap ONV: onvansertib.

We tested the combination of navitoclax with onvansertib on the different mEOC cells. As shown in Figure 5, the combination was synergic in all four cell lines, as demonstrated by the clear shift to the left in the dose–response curve with the addition of increasing non-toxic concentrations of navitoclax, and confirmed by the BLISS-synergism Analysis.

To corroborate the importance of targeting BCL2L2, we also tested the combination of onvansertib with IS21, a recently reported inhibitor of Bcl2, Bcl-xl, and Mcl1 [42,43]: there was mainly an additive effect (Supplementary Figure S3). These data support the fact that BCL2L2 inhibition could be important for the synergistic effect of the onvansertib/navitoclax combination.

**Figure 6:**
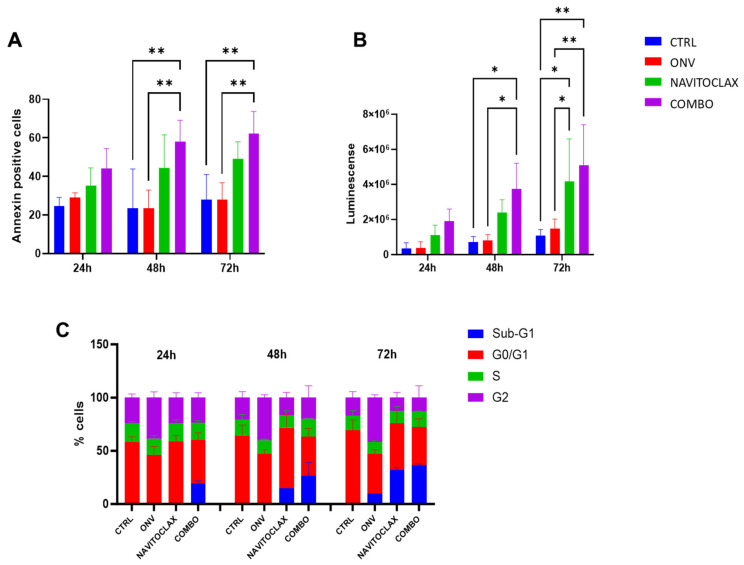
Molecular investigation of the combination in EFO27. Cells were treated with onvansertib 75 nM, navitoclax 1.25 μM, or their combination. At the times indicated, cells were analyzed for Annexin V positivity (**A**), caspase 3/7 activity (**B**), and cell cycle perturbation (**C**). Representative histograms show the percentages of Annexin V-positive cells, analyzed by flow cytometry (**A**), and luminescence signals corresponding to caspase 3/7 activation (**B**). Bar graphs display the quantification of cells in different cell cycle phases (**C**). Data are presented as the mean ± SD of three independent experiments.Ctrl: control; Onv: Onvansertib; *: *p* < 0.05; **: *p* < 0.01.

To investigate the molecular mechanisms underlying this synergistic effect of navitoclax and onvansertib, we examined the induction of apoptosis and the cell cycle perturbation in cells treated with the single drugs (onvansertib 75 nM and navitoclax 1.25 µM) and their combination at different time points in EFO27 cells. Apoptosis was detected with Annexin V positivity and evaluating caspase enzymatic activity (Figure 6A,B). Onvansertib did not induce apoptosis compared to the control cells at 24, 48, and 72 hrs, while navitoclax alone or combined with onvansertib induced apoptosis; with the combination, the induction of apoptosis seemed higher, even though there was no significant difference from the navitoclax single agent.

The cell cycle analysis clearly showed a subG1 peak at 24 h, suggesting that cells treated with the combination had undergone apoptosis, while the percentage of cells blocked in G2 after onvansertib treatment was higher at this time point, as already reported [30], but there was no clear perturbation of the cell cycle with navitoclax. At 48 h, the proportion of cells in the G2 phase was higher after onvansertib and this persisted at 72 h and was accompanied by a subG1 peak; navitoclax caused a subG1 peak that further increased at 72 h. The combination caused a significant increase in subG1 compared to the control (*p* < 0.0001) and onvansertib (*p* < 0.0001) treatments at 24 h and 48 h, and further increased at 72 h (Figure 6C).

## 3. Discussion

mEOC presents therapeutic challenges due to its resistance to standard chemotherapy [44,45]. Therefore, new therapeutic targets are needed for advanced mEOC to improve patients’ survival. We employed a CRISPR/Cas9 screening approach to identify potential new targets interfering with cell growth/survival and to screen for genes with synthetic lethality in PLK1 inhibition.

PLK1 is a conserved Ser/Thr kinase primarily involved in cell cycle regulation, particularly during the G2/S and M phases, where it controls mitosis, centrosome maturation, spindle assembly, and cytokinesis [46]. In addition to controlling cell cycle progression, PLK1 is involved in the DNA damage response [47], autophagy [48], apoptosis [49], and cytokine signaling [50]. Its overexpression has been observed in many cancers, and promotes proliferation, transformation, and epithelial-to-mesenchymal transition [51]. We have already reported that PLK1 represent a new therapeutic target in mEOC as its inhibition with both esiRNA and onvansertib was cytotoxic in an mEOC cell line in vitro and showed antitumor activity alone and in combination with chemotherapy in both high-grade serous OC and mEOC models [28,30].

We used a screening library composed of 31,324 sgRNAs targeting 3015 genes involved in apoptosis and related to cancer in EFO27/Cas9 cells in two experimental settings: following the infection of the library to identify genes interfering with cell growth/survival, and in combination with onvansertib treatment. In the first case, we identified 12 genes, *ZC2HC1C*, *RPA2*, *KIN*, *TUBG1*, *SMC2*, *CDC26*, *CDC42*, *HOXA9*, *TAF10*, *SENP1*, *MRPS31*, *COPS2*, whose interference caused cell death. Among these genes, to begin with only *KIN17* and *SENP1* were selected for further validation as they showed different roles in the development and maintenance of different tumors, but no data have been reported for mEOC. KIN17 is a DNA- and RNA-binding protein that is highly expressed in various human cancers. It plays diverse roles in DNA replication, damage repair, cell cycle regulation, and RNA processing [52,53,54,55]. KIN17 is also linked to cancer cell proliferation, migration, invasion, and cell cycle control through pathways such as p38 MAPK [56], NF-κB–Snail [57], and TGF-β/Smad2 [58]. Importantly, KIN17 knockdown has been shown to enhance tumor cell sensitivity to chemotherapy. Elevated KIN17 expression has been observed in breast cancer [59], where it is associated with poor clinical outcomes, including lower overall survival, reduced relapse-free survival, decreased distant metastasis-free survival, and shorter post-progression survival [60]. In colorectal cancer, KIN17 is upregulated in patients with lymph node metastasis [61]. Similarly, KIN17 expression is significantly higher in cervical cancer compared to normal tissue, and its levels correlate with the severity of cervical lesions [62]. In ovarian serous adenocarcinoma, KIN17 is markedly increased in tumor tissues compared to normal tissues [63]. Additionally, KIN17 is highly expressed in non-small-cell lung cancer and hepatocellular carcinoma [64]. SENP1 was selected for further study following our CRISPR/Cas9 screening due to its well-established role in promoting tumor survival and therapy resistance across a variety of cancers. SENP1 overexpression has been linked to enhanced cell viability, proliferation, invasion, metastasis, stemness, angiogenesis, altered metabolism, and drug resistance [65]. Additionally, it plays a key role in resistance to apoptosis, mediated through pathways involving NF-κB, BCL2 family proteins, and the deSUMOylation of target proteins [66]. In colorectal cancer (CRC), SENP1 contributes to drug resistance by stabilizing HIF-1α, thereby enhancing survival, migration, and metabolic adaptation under stress conditions [67,68]. Given their significant role in cancer progression, particularly in colorectal cancer, which shares similarities with mEOC [69], we chose to further investigate KIN17 and SENP1 in the context of mEOC to better understand their contribution to this challenging cancer subtype.

In EFO7, the downregulation of *KIN17* with esiRNA led to a significant reduction in cell viability, but there were no significant effects in OCM.72. Targeting SENP1 through genetic targeting or using Momordin Ic significantly overcame platinum resistance in ovarian cancer [70]. In our study *SENP1* downregulation resulted in a reduced cell viability in EFO27 and OCM.72 cells. In TOV2414, we were unable to downregulate either gene. The cytotoxicity of a commercially available SENP1 inhibitor (Momordin Ic) was tested in the mEOC cells and it was active at a µM concentration.

The second part of our screening, which involved treatment with onvansertib, identified three genes, *JUND*, *CARD9*, and *BCL2L2*, with possible synthetic lethality with onvansertib. JUND, a member of the AP-1 transcription factor family, modulates tumor angiogenesis, differentiation, and proliferation, and is downregulated in chemotherapy-sensitive ovarian cancer patients [71]. CARD9, a key regulator of caspase activation and NF-κB signaling, was abnormally upregulated in ovarian cancer tissues and cells, where its knockdown led to reduced cell proliferation and enhanced cisplatin sensitivity [72]. BCL2L2 is a pro-survival factor from the BCL-2 family [73]. Of the three genes, we could validate *CARD9* and *BCL2L2*, which, when knocked down with esiRNA, increased onvansertib cytotoxicity.

In order to translate these data to an in vivo application, we looked for commercially available inhibitors, and as no specific inhibitors of BCL2L2 are available yet, and we focused on navitoclax, which is a pan-BCL2 inhibitor and inhibits BCL2, BCL-XL, BCL-W [74]. Navitoclax had significant antitumor activity in patients with myelofibrosis in the phase 2 REFINE trial (NCT03222609) and is undergoing a phase 3, double-blind, placebo-controlled, multicenter, international study (TRANSFORM-1) evaluating its safety and efficacy in combination with ruxolitinib in JAK2 inhibitor-naïve adults with myelofibrosis [42,43].

Considering that navitoclax is a pan-inhibitor, we also tested co-treatment with onvansertib and IS21, which targets BCL2, BCL-XL, and MCL-1. However, we did not observe the same level of synergism, supporting the important role of inhibiting BCL2L2 in the synergistic effect.

We tried to clarify the molecular mechanisms of the synergism and found that the onvansertib/navitoclax combination resulted in cell death through apoptosis, as demonstrated by Annexin V induction, caspase activation, and the appearance of a subG1 peak in cell cycle perturbation studies.

Navitoclax has been shown to potentiate the cytotoxicity of BI2536 (a PLK1 inhibitor) in 2D and 3D models of small-cell lung cancer, being able to overcome mitotic slippage and favor apoptosis [75]. Mitotic slippage is a mechanism by which cells are arrested in G2 exit mitosis without dividing [76]. Our previous data showed that, in EFO27 cells, onvansertib treatment caused the cells to enter mitosis, but not to undergo chromosome segregation and cytokinesis, leading to the formation of polyploid and multinucleated cells [28]. In this case, the combined treatment with navitoclax may strongly drive cells that have undergone mitotic slippage toward apoptosis, and time lapse experiments are under way to address this point.

The combination of volasertib, another PLK1 inhibitor, and ABT199 (inhibitor of BCL2) has recently been shown to be synergistic in several models of double-hit lymphoma (DHL), an aggressive subgroup of B cell lymphoma, both in vitro and in vivo, where the eradication of tumors in validated models of DHL was observed [77]. However, the underlying mechanism responsible for this synergist effect was not clarified. Similar data were obtained in T-cell acute lymphoblastic leukemia (T-ALL), in which the combinations venetoclax/volasertib and venetoclax/onvasertib resulted in a synergistic interaction [78], and the mechanisms underlying the efficacy of the combination included the upregulation of BCL2 members (BL2L13 and NOXA) greatly favoring apoptosis [79].

Despite the results of our CRISPR/Cas9 screening and subsequent validation experiments, several limitations should be taken into account. First, the use of cell lines (EFO27, OCM.72, TOV2414) presents a limitation, as these models may not fully represent the genetic and phenotypic heterogeneity of mEOC. Therefore, while our findings demonstrate significant cytotoxic effects in vitro, these data need to be confirmed in an in vivo setting. In addition, although we successfully validated SENP1 targeting in EFO27 and OCM.72 cells, we were unable to observe this effect in TOV2414 cells, highlighting cell line-specific differences; similar considerations also apply for KIN17.

In summary, while our study identifies potential new therapeutic targets and combinations for use in mEOC, further research is necessary to validate these findings in additional relevant models to foster their clinical translatability.

## 4. Material and Methods

### 4.1. Cell Lines and Drug Treatment

The mEOC cell lines used, MCAS, EFO27, TOV2414 and OCM.72, were obtained from the American Type Culture Collection (ATCC, Manassas, VA, USA) and they were authenticated by the authors in the last 6 months, except for OCM.72, which was kindly supplied by Taylor S. (University of Manchester, 555 Wilmslow Road, Manchester, UK). MCAS cells were grown in MEM with 20% FBS and 1% glutamine, EFO27 cells in RPMI1640 with 10% FBS and 1% glutamine, TOV2414 cells in OSE medium with 10% FBS and 1% glutamine, and OCM.72 cells in a 50:50 mix of Nutrient Mixture Ham’s F12 and Medium 199, supplemented with 5% FBS, 2 mM glutamine, 100 U/mL penicillin, 100 U/mL streptomycin, and 10 mM HEPES at pH 7.4; the medium also contained 20 μg/mL insulin, 0.01 μg/mL EGF, 0.5 μg/mL hydrocortisone, 10 μg/mL transferrin, and 0.2 pg/mL triiodothyronine. HEK293 cells were grown in DMEM medium, with 10% FBS, and 1% of glutamine. All cell lines were maintained in a humidified 37 °C incubator with 5% CO_2._

Onvansertib (kindly provided by Cardiff Oncology, San Diego, LA, USA), IS21 (ChemSpace, Riga, Latvia), Navitoclax (Selleckchem, Houston, TX, USA), and SENP1 inhibitor Momordin Ic (Merck SML3435) were dissolved in DMSO at the concentration of 20 mM, stored at −20 °C, and diluted to their final concentration just before use.

For cytotoxicity experiments, mEOC cells were seeded at a concentration of 30.000 cells/mL in 96 well plates and after 48 h were treated with the different drugs at the concentrations indicated. Cell viability was evaluated using CellTiterGlo (Promega, Madison, WI, USA) after five days of treatment. Luminescence was recorded on a microplate reader (Glomax-Promega). We used the Bliss model in Combenefit software (version 2.021) to calculate and evaluate drug synergism [80].

### 4.2. Plasmids, Library and Lentivirus Production

The plasmids used were pRSV-REV (Addgene #12253) pMD2G (Addgene #12259) and pMDGL/pRRE (Addgene #12253), LentiBlastCas9 (Addgene #52962), and PXPR_011 (Addgene #59702), purchased from Addgene (Watertown, MA, USA). For screening, we used the Lentiviral Bassik Human CRISPR Deletion Library—Apoptosis and Cancer (Addgene #101926), composed of 31,324 sgRNAs, targeting 3015 genes, with about 10 sgRNAs targeting each gene. The library was amplified as described in the Zhang Lab protocol [81].

The Lentiviral Bassik Human CRISPR Library was amplified in bacteria (ElectroMAX Stbl4 cells; Invitrogen, Carlsbad, CA, USA). To produce lentiviral particles, HEK293T cells were transfected 24 h after seeding using Lipofectamine2000 (Life Technologies, Monza, Italy), following the manufacturer’s instructions, with a mix of the transfer plasmid of interest and the packaging/envelope plasmids: pRSV-Rev, pMDLg/pRRE, and pMDG.2. After 48–72 h following transfection, the medium containing the lentiviral particles was harvested and centrifuged and the supernatant was stored at −80° until use.

### 4.3. Generation of Cas9 Over-Expression Mucinous Cells

Cells seeded at the concentration of 50,000 cells/well in a six-well plate were infected with lentiviral particles containing lentiCas9-Blast with 10 µg/mL of polybrene (Merck, Darmstadt, Germany) after 24 h, and after 72 h, blasticidin 5 µg/mL (Gibco, Waltham, MA, USA) was added for cell selection.

To evaluate Cas9 activity, 50,000 cells seeded in a six-well plate with the addition of 10 µg/mL of polybrene were infected with the lentiviral plasmid PXPR_011 carrying the GFP sequence, the gRNA targeting GFP, and a puromycin resistance gene. After 72 h, the infected cells were treated with 1 µg/mL puromycin to select infected cells, and after five days, GFP expression was analyzed using fluorescence-activated cell sorting (FACS), quantifying the percentage of GFP-negative cells. Cas9 activity is directly correlated with GFP-negative cells [35].

### 4.4. Western Blot Analysis

Cell pellets were lysed for 30 min in ice-cold whole-cell extract buffer (50 mM TrisHCl pH 7.4) 250 mM NaCl, 0.1% Nonidet NP40, 5 mM EDTA, 50 mM NaF, and a protease inhibitor cocktail (Sigma-Aldrich, St. Louis, MO, USA). Lysates were cleared by centrifuging at 12,000 rpm for 15 min and the protein concentration was determined using a Bio-RadAD assay kit (BIO-RAD Laboratories S.r.l (BioRad, Hercules, CA, USA). Cell lysates (50–40 µg) were resolved on 10–12% SDS-PAGE (polyacrylamide gel electrophoresis) gels. Proteins were then transferred into nitrocellulose membranes (Merck Millipore, Burlington, MA, USA). Immunoblotting was carried out with anti-Cas9 (Cell Signaling, Danvers, MA, USA, #19526), a KIN17 antibody (Santa Cruz, Santa Cruz, CA, USA, sc-32769), and anti-HSP90 (Santa Cruz, sc-69703) antibodies. The secondary antibodies conjugated with horseradish peroxidase (HRP), anti-rabbit #1706515, and anti-mouse #1706516 were purchased from BIO-RAD Laboratories S.r.l. HRP substrate (ECL Western blotting Detection, Amersham-Life Science, Buckinghamshire, UK) was added and the signal was detected with the Odyssey Fc instrument (Li-COR, Lincoln, NE, USA).

### 4.5. CRISPR/Cas9 Screening

We aimed at 500× coverage and, considering an MOI of 0.3, we infected 600 × 10^6^ EFO27/Cas9 cells with puromycin (1 µg/mL) 72 h after infection. After 6 days (time point-T0) of selection with puromycin, 15 × 10^6^ cells were harvested for NGS sequencing, and 30 × 10^6^ were split as untreated or treated cells (150 nM onvansertib). After 7 days (time point 1-T1), 15 × 10^6^ cells for each condition were harvested for NGS. Genomic DNA was extracted at each time point using the QIAmp DNA Blood Maxi kit (QIAGEN, Milano, Italy).

To prepare the sgRNA sequencing library, the integrated sgRNA-encoding constructs were PCR-amplified using the Bassik Lab protocol [82]. PCR products were cleaned using AMPure XP beads (Beckman Coulter, Brea, CA, USA) following the manufacturer’s protocol. Product quality was assessed using the 4200 Tapestation High Sensitivity D1000 (Agilent Technologies, Santa Clara, CA, USA) and the Qubit™ High Sensitivity dsDNA Assay Kit (Invitrogen). Libraries were prepared at 1.8 pM with 10% PhiX and sequenced on a NextSeq 500 (Illumina, San Diego, CA, USA) using a 150-cycle High Output Cartridge.

### 4.6. NGS Data Analyses

CRISPR-seq data were processed using the MAGeCK pipeline [36]. Raw FASTQ files were first aligned to the corresponding sgRNA library using the MAGeCK count with normalization based on negative control sgRNAs. Samples were untreated or treated. Differential gene essentiality was calculated using the MAGeCK test to compare time points and conditions. For post-processing and downstream analysis, we used the MAGeCKFlute R package (version v0.5.9.5) [37]. For genes involved in cell survival selection was based on the FDR calculated at the gene level. For genes with synthetic lethality for onvasertib, the selection was based on the FDR < 0.1 of individual sgRNAs, where differences may reflect varying degrees of sensitivity or interaction with the drug, possibly indicating synergism between onvansertib and these genes. Rank plots were generated to visualize positive and negative selections.

### 4.7. esiRNA Transfection

mEOC cells were seeded at 20,000 cells/mL, and after 24 h were transfected with Lipofectamine2000 reagent (Life Technologies, Monza, Italy) and not-targeting esiRNA (scramble esiRNA) (Merck SIC001) and a specific esiRNA: KIN (Merck EHU138951); SENP1 (Merck EHU160431), JUND (Santa Cruz, sc-35728), CARD9 (Merck EHU139641) and BCL2L2 (Merck EHU058421). The concentrations of esiRNA used were 70 nM for KIN, SENP1, and CARD9, 50 nM for JUND, 40 nM for BCL2L2, and 50 nM for scramble esiRNA.

To study the effect of the combination of onvansertib and downregulation of *CARD9*, *JUND*, and *BCL2L2*, the drug was added 24 h after transfection, at the subtoxic dose of 150 nM. After 72 h from transfection cell viability was assessed using CellTiter Glo reagent (Promega).

### 4.8. RT-PCR

Total RNA was extracted using a Maxwell^®^ RSC simplyRNA kit (Promega) and reverse transcribed to cDNA with a HighCapacity cDNA Retrotranscription Kit (Applied Biosystems, Waltham, MA, USA). Optimal primer pairs were identified for selected genes (Supplementary Table S1). cDNA was amplified by real time RT-PCR (ABI-7900, Applied Biosystems) with the SYBR Green technique. Actin was used as the internal control. Relative quantification of mRNA was carried out using the Δ∆Ct method.

### 4.9. Cell Cycle/Annexin V

Cells were seeded at 20,000 cells/mL in a 12 well-plate. After 72 h, cells were treated and at different time points (24, 48, 72 h) detached and stained for Annexin V using FITC Annexin V Apoptosis Detection Kit with P (Biolegend, San Diego, CA, USA). For cell cycle analysis, a flow cytometric analysis of DNA content was performed at different times after treatment. Cells were fixed in ice-cold 70% ethanol, washed in PBS, suspended in 1 mL of PBS containing 25 μg/mL of propidium iodide and 12.5 μL of RNase (1 mg/mL), and stained for 6 h at room temperature.

### 4.10. Caspase Activity

Caspase-3/7 activity was measured with an enzymatic assay. Cells were seeded in 96-well plate at concentration of 30,000/mL, and treated after 72 h. After 24, 48, and 72 h, caspase activity was measured using a plate reader (Glomax, Promega). The activity of each sample was examined in triplicate. Activity was expressed as the registered luminescence during different intervals

### 4.11. Statistical Analysis

Statistical analyses were performed with GraphPad Prism Software 10.1.2 (GraphPad, La Jolla, CA, USA). Each experiment was replicated two to five times.

## 5. Conclusions

These findings suggest that onvansertib/navitoclax can offer a new therapeutic strategy for mEOC. The synergistic effect of the combination will allow the use of lower doses of both compounds, greatly limiting potential side effects. These data need to be confirmed in in vivo models of mEOC which, if positive, will provide a strong rationale for clinical trials in patients with mEOC.

## Data Availability

The data presented in this study are available on request from the corresponding author.

## Supplementary Materials

The following supporting information can be downloaded at: https://www.mdpi.com/article/10.3390/ijms26020472/s1.
