# Supplementary material for: Onvansertib and Navitoclax Combination as a New Therapeutic Option for Mucinous Ovarian Carcinoma
Source: Int J Mol Sci. 2025 Jan 8;26(2):472. doi: 10.3390/ijms26020472 (PMC11765470; doi:10.3390/ijms26020472)
Supplement: Supplementary file 1 [file ijms-26-00472-s001.zip › ijms-3306338-supplementary.pdf]

Onvansertib and Navitoclax Combination as a New Therapeutic Option for Mucinous Ovarian Carcinoma

Serena Petrella [1], Marika Colombo [2], Mirko Marabese [2], Chiara Grasselli [3], Andrea Panfili [3], Michela Chiappa [1], Valentina Sancisi [4], Ilaria Craparotta [5], Maria C. Barbera [5], Giada A. Cassanmagnago [5], Marco Bolis [5] and Giovanna Damia [1,*]

[1] Laboratory of Gynecological Preclinical Oncology, Department of Experimental Oncology, Istituto di Ricerche Farmacologiche Mario Negri IRCCS, 20156 Milan, Italy
[2] Laboratory of Molecular Pharmacology, Department of Experimental Oncology, Istituto di Ricerche Farmacologiche Mario Negri IRCCS, 20156 Milan, Italy
[3] Laboratory of Immunopharmacology, Department of Experimental Oncology, Istituto di Ricerche Farmacologiche Mario Negri IRCCS, 20156 Milan, Italy
[4] Translational Research Laboratory, Azienda Unità Sanitaria Locale-IRCCS di Reggio Emilia, 42123 Reggio Emilia, Italy
[5] Computational Oncology Unit, Department of Experimental Oncology, Istituto di Ricerche Farmacologiche Mario Negri IRCCS, 20156 Milan, Italy
* Correspondence: giovanna.damia@marionegri.it

**KIN FORWARD**	CATCAGAGACAACTATTGCTGGC
**KIN REVERSE**	AGTGCCAAAGCGTCTCCTGAGA
**SENP1 FORWARD**	TCATCATCTGGATCTGCCAGC
**SENP1 REVERSE**	ACAGAGTGGTGATGATGGGG
**JUND FORWARD**	ATCGACATGGACACGCAGGAGC
**JUND REVERSE**	CTCCGTGTTCTGACTCTTGAGG
**CARD9 FORWARD**	CTGAGGCCATGTCGGACTAC
**CARD9 REVERSE**	CCACTTTCCGTTTGCGGATG
**BCL2L2 FORWARD**	AAGTGCAGGAGTGGATGGTG
**BCL2L2 REVERSE**	TGGCCCTGGACTTTCACTTG

Supplementary Table S1. Primers cDNA.

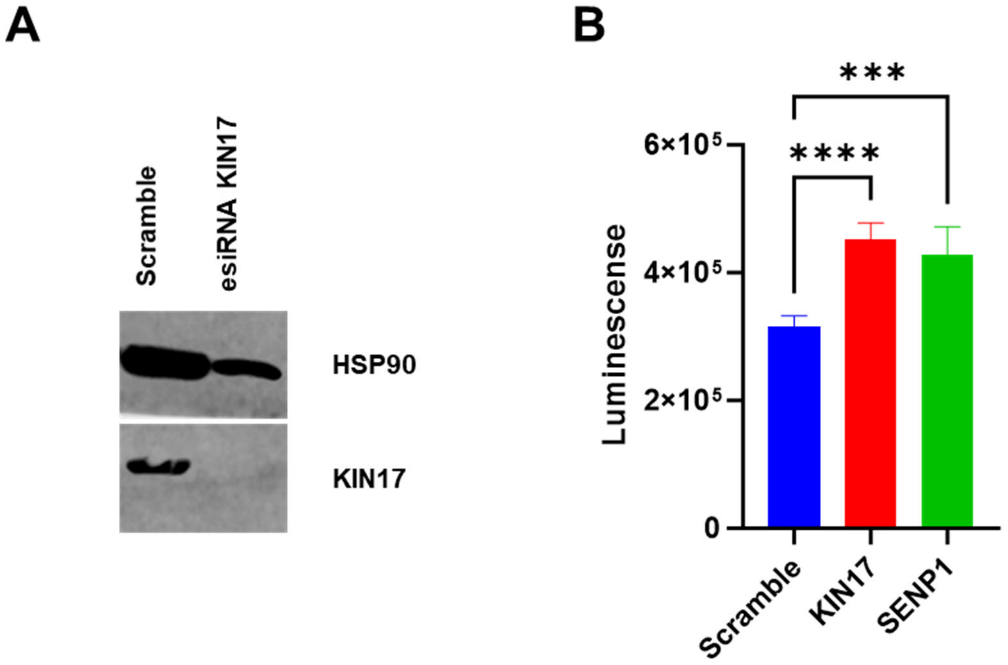

Supplementary Figure S1. After 72h from transfection proteins were extracted from EFO27 cells transfected with esiRNA targeting KIN17 and not targeting siRNA, and analysed to evaluate the expression of targeted protein (A), the result shows a lack of KIN17 protein in cells transfected with KIN17 esiRNA. In EFO27 were evaluated caspase 3/7 activation after72h from transfection (B). A significant activity was observed in cells transfected with KIN17 and SENP1 esiRNA compared to cell transfected with not targeting siRNA. Data are the mean ± SD of at least two independent experiments and are expressed as luminescence signals. ***: $p \leq 0.001$; ****: $p \leq 0.0001$.

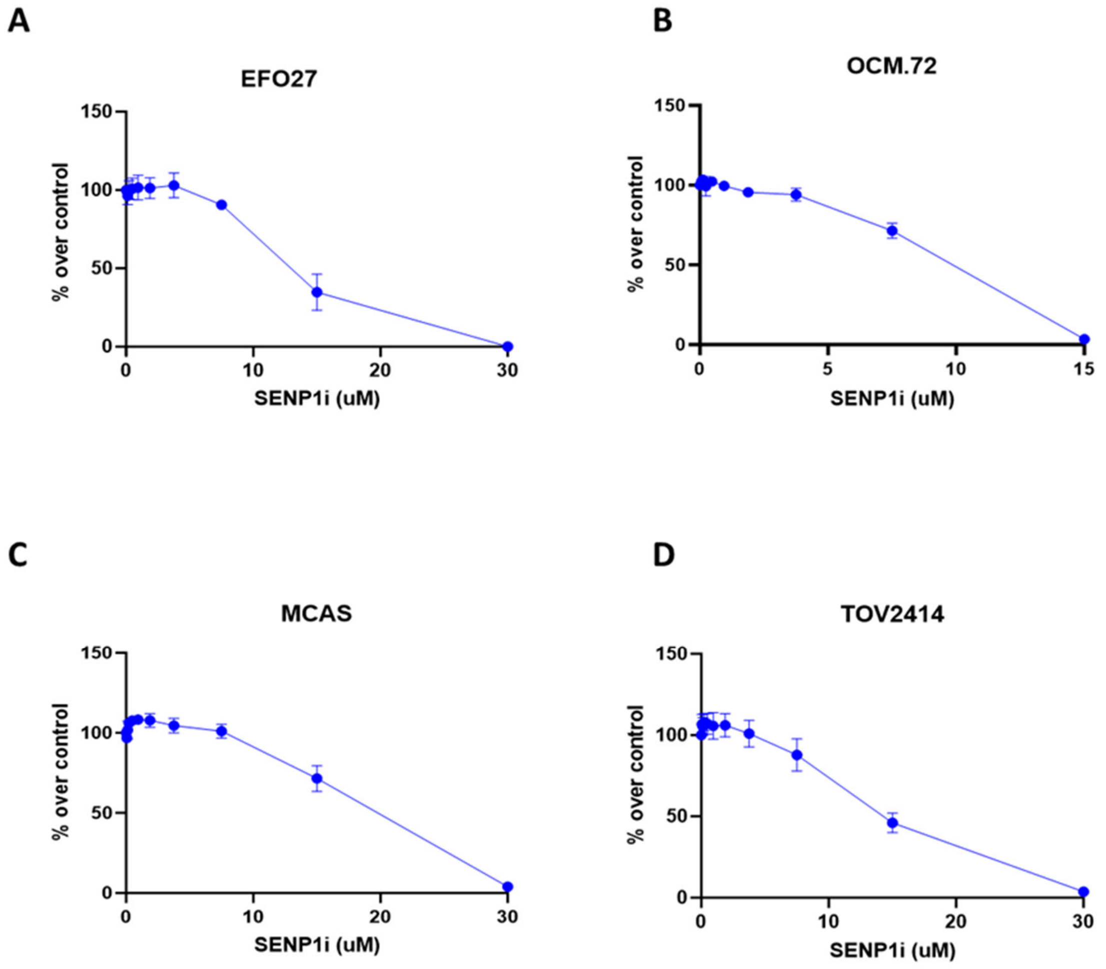

Supplementary Figure S2. Momordin IC cytotoxic activity in mEOC cell lines. Momordin IC dose response curves in EFO27 (A), OCM.72 (B), MCAS (C), and TOV2414 (D) cells. Data are the mean ± SD of at least two independent experiments and are expressed as % of control untreated cells.

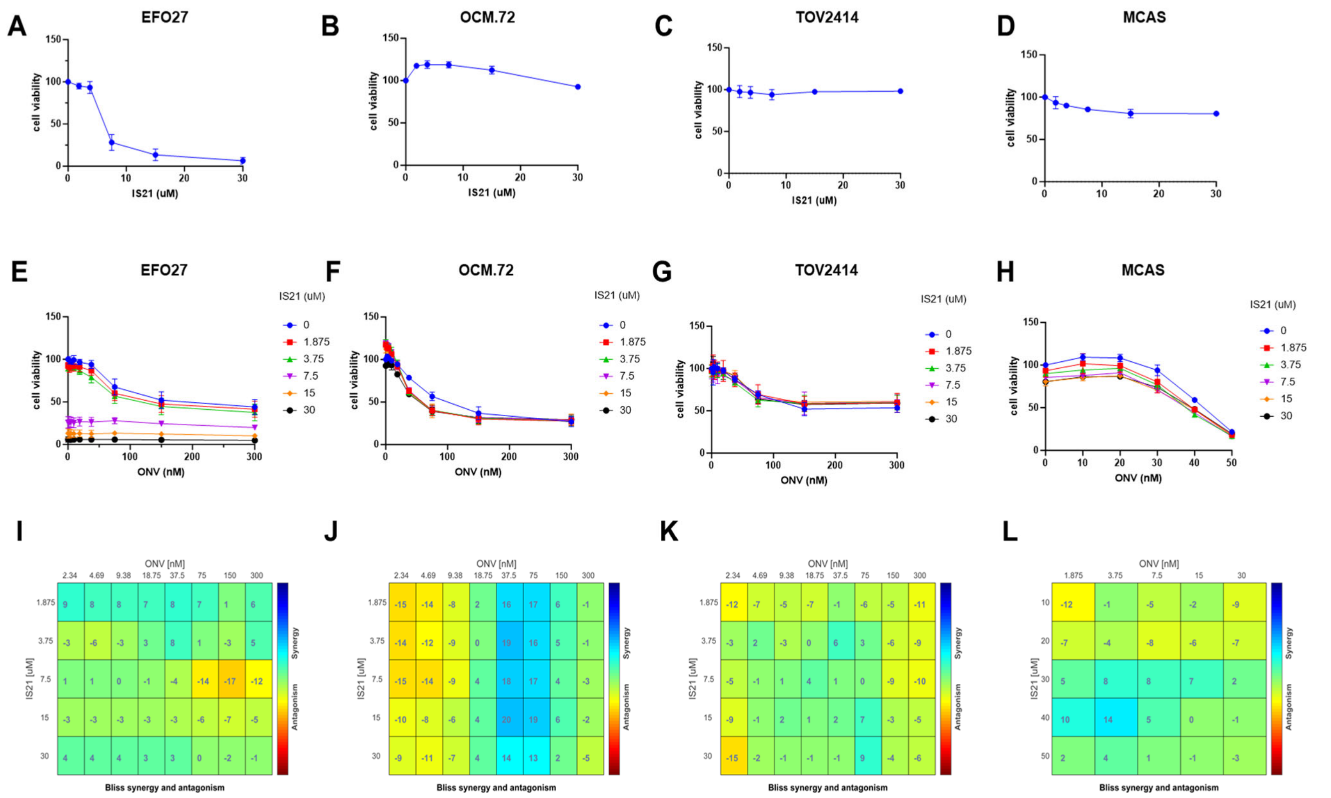

Supplementary Figure S3. Combination of IS21 and ONV on mEOC cells All the available mEOC cell lines were tested with the combination. **A-D** The only IS21 displayed an effect only in EFO27 (A). **E-H** The all mEOC cell lines treated with the combination didn't show a synergistic effect, notable from curves representing cell viability at different concentration of ONV and IS21, in which the blue curve is the only ONV treatment, and the other curves are the combination with different concentration of IS21. Data are the mean ± SD of at least two independent experiments and are expressed as % of control untreated cells. **I-J-K-L** Bliss Synergy Heatmap didn't report a strong synergy effect, that would be indicated by blue squares.
